# Structural Engineering of Edible Oleogels: From Molecular Assembly to Functional Food Applications

**DOI:** 10.3390/gels12070649

**Published:** 2026-07-20

**Authors:** Jun An, Feiyan Yang, Liyou Zheng, Tao Yang

**Affiliations:** 1 College of Food Science and Engineering, Central South University of Forestry and Technology, Changsha 410004, China; anjunyang@126.com; 2 Shenzhen Lam Soon Edible Oils Co., Ltd., Shenzhen 518067, China; 3 School of Liquor and Food Engineering, Guizhou Rosa Roxburghii Research Institute/Guizhou Technical Innovation Center of Industry, Guizhou University, Guiyang 550025, China; 4 School of Biological and Food Engineering, Anhui Polytechnic University, Wuhu 241000, China; zhengliyou@ahpu.edu.cn

**Keywords:** oleogels, structural regulation, gelling agents, applications

## Abstract

Edible oleogels have emerged as promising alternatives to conventional solid fats. They convert liquid oils into semi-solid structures while reducing saturated and trans fatty acid intake. Despite rapid growth in this field, their performance is not governed by a single factor but by coupled effects of oil composition, oleogelator properties, intermolecular interactions, and processing history. In recent years, substantial progress has been achieved in understanding the formation mechanisms, structural regulation strategies, and food applications of oleogels. This review systematically summarizes the key factors influencing oleogel formation and performance, including oil phase composition, gelator type and concentration, molecular interactions, and processing parameters. Emerging structural modulation approaches, such as multi-component oleogelators, hybrid biopolymer systems, and advanced processing technologies, are critically discussed with respect to their effects on crystallization behavior, network architecture, rheological properties, thermal stability, and oil-binding capacity. Particular emphasis is placed on the hierarchical structure–function relationships linking molecular assembly and network organization to macroscopic functionality, digestibility, and bioactive compound delivery. Recent applications of oleogels in bakery products, meat analogs, dairy alternatives, confectionery products, and frying systems are also reviewed. Finally, current challenges and future opportunities related to next-generation oleogelators, hierarchical structural regulation, intelligent responsive systems, and industrial-scale manufacturing are discussed. This review provides a comprehensive framework for understanding the structural engineering of edible oleogels and offers insights into the rational design of healthier and more functional lipid-based food systems.

## 1. Introduction

The rising prevalence of obesity, cardiovascular disease, and metabolic disorders has intensified scrutiny of dietary fat intake, particularly the excessive intake of saturated and trans fatty acids [1,2]. In response, global health authorities have called for their elimination or strict limitation [3]. Yet reformulating fat-rich foods remains difficult. Solid fats are not easily replaced without compromising texture and sensory structure.

Oleogels have emerged as a viable structural alternative. They convert liquid oils into semi-solid systems without hydrogenation, thus avoiding trans-fat formation while reducing saturated fat content [4,5,6]. Additionally, oleogels offer tunable physicochemical properties, enabling the modulation of hardness, plasticity, and thermal behavior while maintaining desirable functional attributes [7,8].

Over the past decade, research efforts in this field have primarily focused on three aspects: the development of novel oleogelators, the optimization of gelation and processing techniques, and the exploration of food applications. A wide range of structuring agents, including natural waxes, monoglycerides, fatty acids, proteins, polysaccharides, and hybrid systems, have been employed to construct three-dimensional (3D) networks capable of immobilizing liquid oils [9,10]. Meanwhile, diverse preparation strategies such as direct dispersion and indirect methods have been developed to tailor oleogel properties for specific functional requirements [11,12].

Despite these advances, current research remains largely empirical and fragmented, with most studies focusing on specific systems and yielding findings that are difficult to generalize. Moreover, mechanistic understanding is still incomplete because molecular interactions, crystallization pathways, and processing history are often investigated independently, leaving their coupled effects insufficiently understood. In particular, the relationships among molecular interactions, crystal engineering, processing-induced structural evolution, and functional properties including rheological behavior, oil-binding capacity, digestibility, and bioactive delivery have not been systematically integrated into a unified framework. This lack of structure–function understanding restricts the rational design and scalable application of oleogel systems in the food industry.

Therefore, this review aims to provide a comprehensive and critical overview of recent advances in edible oleogels from a structural engineering perspective. Unlike previous reviews that primarily focus on formulation strategies or application cases, this work emphasizes the hierarchical organization of oleogels from molecular assembly to crystal network formation and macroscopic functionality. Specifically, we integrate (i) structural regulation mechanisms involving oil phase characteristics, oleogelator chemistry, and processing conditions; (ii) hierarchical assembly pathways governing network formation; and (iii) structure–function relationships linking microstructure to physicochemical and nutritional performance. Finally, current challenges and future perspectives are discussed, including clean-label design, intelligent formulation strategies, precision nutrition, and industrial-scale production, with the aim of advancing the rational design of next-generation oleogel-based food systems.

## 2. Factors Influencing the Structural and Physicochemical Properties of Oleogels

**Figure 1:**
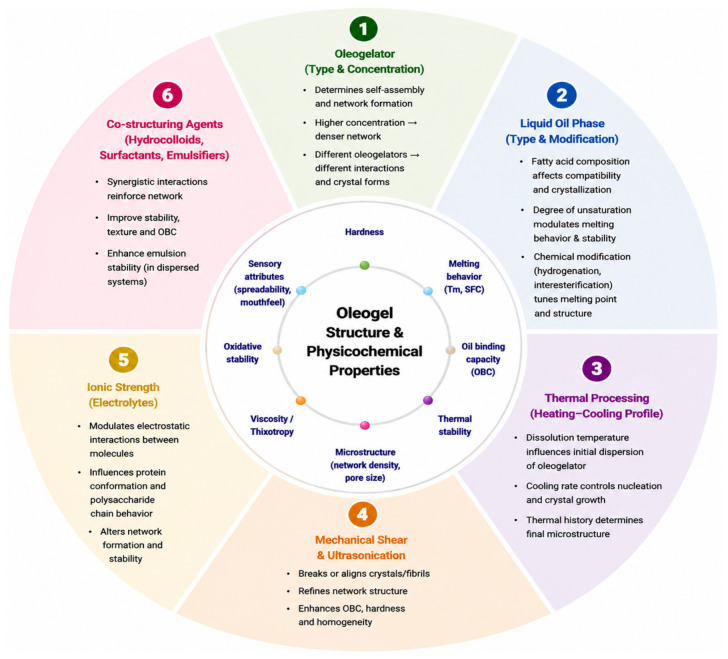
Influence of processing parameters on characteristics of oleogels.

The structural architecture and physicochemical properties of oleogels are governed by a range of interrelated formulation and processing parameters [7,8,13,14] (Figure 1). These factors do not act independently. Instead, they jointly regulate crystallization behavior, network development, and macroscopic functionality. To organize this complexity, the discussion is divided into three key determinants: (i) liquid oil phase, (ii) oleogelators, and (iii) processing conditions. This classification is not merely descriptive. It reflects the hierarchical contribution of each component to structure formation.

Within this framework, molecular-level interactions can be more directly linked to bulk gel properties. Such a perspective helps move beyond the empirical formulation toward more rational design of oleogel systems for targeted applications. A more detailed comparison of individual effects is summarized in Table 1.

### 2.1. Selection and Modification of the Oil Phase

The type and physicochemical properties of the liquid oil phase are key determinants of oleogel structure, as they directly influence gelation behavior, network formation, and final functional performance [7,15]. Variations in fatty acid composition, molecular structure, and polarity modulate bulk oil properties such as viscosity, melting behavior, and intermolecular interactions with oleogelators, thereby affecting the assembly and stability of the three-dimensional gel network [16,17,18].

Among these factors, the fatty acid profile of the continuous lipid phase plays a central role. In particular, the degree of unsaturation strongly governs crystallization kinetics and network architecture [11,18]. The effect of unsaturation on oleogel strength is not monotonic and should not be interpreted independently of the oleogelator. Unsaturated oils exhibit lower melting points and greater molecular mobility, which can improve oleogelator dissolution and facilitate the formation of fine, interconnected networks when oil–gelator compatibility is favorable. However, the same structural disorder may weaken oil-phase packing and reduce rigidity. By contrast, saturated acyl chains favor ordered packing and stronger van der Waals interactions and may therefore reinforce crystal-mediated networks. Accordingly, the final mechanical response is governed by the combined effects of fatty-acid composition, gelator solubility, co-crystallization, crystal morphology, and processing history rather than by unsaturation alone [11,16].

The influence of fatty acid composition is further reflected in the performance differences among various edible oils. Oils rich in monounsaturated fatty acids (MUFAs), such as olive oil, and those enriched in polyunsaturated fatty acids (PUFAs), such as sunflower, soybean, chia, and flaxseed oils, often exhibit distinct gelation behaviors and thermal transitions. MUFA-rich oils, such as olive and high-oleic sunflower oils, generally provide an intermediate balance between molecular mobility, oxidative stability, and gelator compatibility. PUFA-rich oils, including flaxseed and chia oils, remain more fluid at low temperature and may promote rapid gelator diffusion and the development of fine crystal networks; nevertheless, their high degree of unsaturation can lower thermal resistance and increase oxidative susceptibility. Therefore, PUFA-rich systems may show high oil-binding capacity with compatible crystalline oleogelators, whereas MUFA-rich systems often provide greater oxidative and storage stability. These trends remain gelator-dependent and should be interpreted together with cooling history and minor oil components. For instance, the flaxseed- and chia-oil oleogels exhibited higher hardness and up to 48% greater oil-binding capacity (OBC) than the corresponding sunflower-, soybean-, and sesame-oil formulations [15]. In addition, variations in fatty acid composition significantly affect thermal stability, with differences in PUFA/SFA ratios influencing melting temperatures and phase transition behavior in wax-based oleogels [16]. Furthermore, a previous study demonstrated that oleogel viscosity and hardness depend strongly on oil type, generally increasing with higher saturation levels [19].

Chemical modification is relevant to oleogel design only when it changes the physicochemical compatibility between the liquid oil phase and the oleogelator. Full hydrogenation substantially increases melting point and solid-fat content, and may convert part of the nominal oil phase into a crystalline hard stock. This hydrogenated fraction should be regarded as a co-oleogelator rather than as a conventional liquid oil phase. Interesterification, particularly enzymatic interesterification, redistributes acyl groups among triacylglycerols without necessarily changing the overall fatty-acid profile. When the modified lipid remains liquid at processing and storage temperatures, the altered triacylglycerol distribution can affect gelator solubility, heterogeneous nucleation, crystal growth, and oil-oleogelator affinity [20,21,22].

Minor oil components also deserve explicit consideration. Phospholipids, free fatty acids, sterols, pigments, endogenous waxes, and oxidation products can modify polarity, interfacial behavior, and heterogeneous nucleation. Their removal during refining may either improve or weaken gelation depending on the oleogelator [23]. Therefore, the oil phase should be selected according to its complete compositional profile and compatibility with the chosen structuring mechanism.

Overall, the physicochemical characteristics of the liquid oil phase, particularly the fatty acid composition, degree of unsaturation, and molecular polarity, play a decisive role in governing oleogel structure and performance. Rational selection and targeted modification of lipid matrices, in combination with appropriate oleogelators, enable the design of oleogels with tailored mechanical strength, thermal stability, and sensory properties for diverse food applications.

**Table 1:** Effect of factors on the physical and functional characteristics of edible oleogels.

Oleogelators	Concentrations (wt%)	Oil Phases	Main Findings	References
High-molecular-weight chitosan	0.8% and 1.0%	Olive oil	A higher chitosan concentration (1%) significantly enhanced the viscoelastic moduli, whereas the oil/water ratio exerted only a limited effect. The oleogels exhibited high oil binding capacity (86–88%), which was not substantially influenced by either chitosan concentration or the oil/water ratio.	[24]
Ovalbumin-proanthocyanidin-sodium alginate conjugate	0.2–1.0%	Sunflower oil	The concentration of ovalbumin-proanthocyanidin-sodium alginate conjugates influenced the crystalline properties and surface microstructure of oleogels, as higher concentrations produced greater crystal density and more uniformly distributed surface protrusions.	[25]
β-Cyclodextrin (β-CD), xanthan gum (XG), and monoglyceride (MG)	XG (0.2%), MG (3%), and β-CD (0%~3.2%)	Camellia oil	Acting as a Pickering stabilizer at the oil/water interface, the β-CD-XG-MG complex significantly improved the gel strength, viscoelasticity, and thixotropic stability of the oleogels.	[26]
Beeswax	5%	Canola oil, peanut oil, and sesame oil	Sesame oil was characterized by smaller crystal sizes, lower oil binding capacity, and reduced hardness than canola oil and peanut oil, suggesting a weaker structural network.	[11]
Monoglyceride stearate and beeswax	0, 2%, 4%, 6%, 8%, 10%	Rice bran oil	The oleogel formulated with a high gelator concentration displayed a more stable crystalline network. Monoglyceride stearate-based oleogels demonstrated enhanced oil binding capacity (89.26 ± 0.12%), hardness (5.195 ± 0.333 26 g) and oxidative stability, resulting from the establishment of a robust polymer network structure.	[1]
Candelilla wax, carnauba wax, rice bran wax, sunflower wax, and beeswax, mono- and diglycerides, and hard fat	Wax-based oleogels were prepared with either 5, 10, or 15% wax in high-oleic sunflower oil, and multi-component oleogels were prepared using a mixture of 5% or 10% wax with mono- and diglycerides or hard fat (15% total structurant)	High-oleic sunflower oil	The addition of hard fat failed to produce cooperative interactions across all waxes, resulting in decreased gel strength. Beeswax-mono- and diglycerides and sunflower wax-mono- and diglyceride gel hardness decreased by day 5 before stabilizing, while the brittleness factor was not significantly impacted.	[27]
Guar gum and rice bran wax	8% and 9%	Walnut oil	The formulation containing 9% rice bran wax exhibited a higher oil-binding capacity than the formulation containing 8% rice bran wax, resulting in a stronger bigel network.	[28]
Candelilla wax	0.75–4.0%	Canola oil	Increasing the candelilla wax contents increased the crystal number (4194 ± 381 to 7646 ± 544) and hardness (6.45 ± 0.30 to 10.4 ± 0.36 N) of fat-tailored oleogels, besides accelerating crystallization.	[29]
Chitosan	1–3%	Olive oil	Increasing chitosan concentration enhanced the stiffness and structural integrity of the oleogels, whereas increasing the chitosan concentration to values higher than 2% did not significantly improve the oil-binding capacity.	[30]
Carnauba wax	7%, 9%, and 11%	Pumpkin seed oil, rice bran oil, and grapeseed oil	Higher wax content (7–11%) increased the firmness and storage modulus (from ∼10^3^ to 10^6^ Pa) of oleogels. Grapeseed oil oleogels showed superior gel integrity and texture.	[31]
Rice bran wax	2.5% and 5%	Sunflower oil and soybean oil	Increasing rice bran wax concentration enhanced firmness and structural consistency, confirming stronger crystalline network formation.	[16]
Rice bran wax, monoglyceride stearate, beeswax, and a mixture of β-sitosterol and γ-oryzanol	7.0%	Macadamia oil	A mixture of β-sitosterol and γ-oryzanol-based oleogels had the highest hardness and oil-binding capacity, and the values of hardness and oil-binding capacity of the four oleogels were in the following order: the mixture of β-sitosterol and γ-oryzanol > monoglyceride stearate > rice bran wax > beeswax.	[32]
Lecithin and hydrogenated lecithin	Glyceryl stearate concentration was fixed at 20%, whereas lecithin or hydrogenated lecithin was varied up to 2.5%	Corn oil	With lecithin, crystallization and melting temperatures were reduced, resulting in less-ordered crystal networks with a lower hardness and oil binding capacity, while with hydrogenated lecithin, the opposite effect was observed. Finally, β-carotene acted as a crystal modifier, increasing the hardness and oil-binding capacity in the presence of lecithin, but decreased these parameters in hydrogenated lecithin-containing and water-filled oleogels.	[33]
γ-Oryzanol/β-sitosterol, γ-oryzanol/triglyceride, monoglycerides, beeswax, beeswax-monoglycerides, and carnauba wax	11%	Iron walnut oil	Iron-walnut-oil oleogel prepared with γ-oryzanol/β-sitosterol had a more stable network structure, excellent hardness at 4 °C (1116.51 g), better antioxidant capacity of 766.50 μmol Trolox equivalents/kg and higher total phenolic content (14.98 mg/kg) than any other experimental iron-walnut-oil oleogel.	[34]
Beeswax, beeswax fractions, and monoesters	6%	Sunflower oil	Oleogels containing 30–50% hydrocarbons exhibited lower oil-binding capacity and firmness than those formulated with 10–20% hydrocarbons. In contrast, the incorporation of 10–20% monoesters improved oleogel firmness, although further increasing the monoester content to 50% reduced this effect.	[35]
Ethyl cellulose, β-sitosterol/γ-oryzanol, and glyceryl monostearate	10%, 15%, and 20%	Coconut oil	Mechanical strength, storage modulus and thermodynamic stability of coconut oil oleogel showed positive dependence on gelling agent dosage.	[36]
Monoglyceride	6% and 10%	Sunflower, chia, flaxseed, soybean, and sesame oils	Both hardness and adhesiveness of the oleogels increased with monoglyceride concentration.	[15]
Glyceryl monostearate, glyceryl monolaurate, glycerol monocaprylate, monoolein, diolein	4–10%	Soybean oil	All the oleogels formed by saturated fatty acid glycerides exhibited a solid-like behavior and were thermally reversible systems, while a higher amount of unsaturated fatty acid glycerides was needed to form oleogels.	[37]
Mono- and diglycerides	10%	High-oleic palm oil	The hardness of the oleogel generally increased with emulsifier addition.	[38]
Carnauba wax, β-sitosterol/beeswax, β-sitosterol/lecithin, and glycerol monostearate	10%	Refined sunflower oil	The β-sitosterol/beeswax oleogel showed the lowest oil loss (0.05%) and a higher hardness (6.37 N) than the commercial margarine reference (3.58 N).	[39]
Carnauba wax	5% or 10%	Palm oil	Oleogels formulated with 10% carnauba wax exhibited significantly improved textural properties, including hardness, stickiness, and tackiness, compared with those containing 5% carnauba wax.	[40]
Beeswax	4%	Camellia oil, sunflower oil, corn oil, and linseed oil	Highly polyunsaturated oleogels induced needle-shaped beeswax crystals with increased phase transition temperatures and denser networks for better stability, and facilitated crystal aggregation especially under low cooling rate.	[41]
Sunflower wax, rice bran wax, candelilla wax, and beeswax	3%	Olive oil	A synergism between sunflower wax and rice bran wax was discovered, as well as between candelilla wax and beeswax. These mixtures displayed an increased hardness, elastic constant, plasticity and oil binding capacity relative to wax oleogels structured by the same concentration of the individual waxes.	[42]
Stigmasterol	1%, 2%, 3%, 4%, 5%, 6%, 7%, 8%, and 9%	Rapeseed oil, olive oil, and flaxseed oil	An increase in stigmasterol concentration was associated with enhanced structural properties of the oleogels.	[43]
Rice bran wax	3%, 5%, 7%, and 9%	Expeller-pressed corn germ oil versus refined corn oil	Rice bran wax could form oleogels in both refined and expeller-pressed corn germ oils at a concentration ≥ 3 wt%. Refined corn oil produced a stronger gel than crude corn oil.	[23]
Ethyl cellulose, mono- and di-glycerides, and a mixture of β-sitosterol + γ-oryzanol	8–15%	Canola oil	Oleogel hardness values in the order of mono- and di-glycerides < ethyl cellulose < β-sitosterol + γ-oryzanol mixture was obtained.	[44]

### 2.2. Selection and Optimization of Structuring Agents

The structural architecture and physicochemical properties of oleogels are primarily governed by the selection and optimization of oleogelators, which serve as the fundamental building blocks for transforming liquid lipids into self-standing semi-solid systems. Based on their gelation mechanisms, oleogelators can be broadly classified into three categories: (i) low-molecular-weight compounds forming crystalline particle networks (e.g., fatty acids, monoglycerides, phytosterols, and natural waxes), (ii) self-assembled fibrillar systems (e.g., 12-hydroxystearic acid), and (iii) biopolymer-based networks (e.g., ethylcellulose, polysaccharides, and proteins) [7,45].

In most systems, single-component oleogelators have been widely used; however, their application is often limited by high gelation temperatures and restricted tunability of structural and functional properties [16]. In contrast, multi-component systems have emerged as a more effective strategy, as synergistic interactions between different oleogelators can significantly reduce gelation temperatures while enhancing texture and structural stability, thereby improving their applicability in food systems [33].

The concentration of oleogelators is a critical parameter that directly determines network density, crystal morphology, and macroscopic mechanical properties. Generally, increasing oleogelator content promotes the formation of more interconnected crystalline or fibrillar networks, leading to enhanced gel strength, hardness, and oil-binding capacity [15,16]. For example, monoglyceride- and wax-based systems exhibit concentration-dependent transitions in rheological behavior, where higher loading levels result in more compact and stable gel networks [46,47]. Similarly, gel strength increases with biopolymer concentration in protein- and polysaccharide-based systems, which arises from strengthened intermolecular interactions including hydrogen bonding [48]. Specifically, when optimized at higher oleogelator concentrations, oleogels exhibit increased hardness values, a phenomenon largely attributed to the proliferation of intermolecular hydrogen bonding that enhances structural integrity [48]. According to a previous study, adjusting the protein concentration (φ) provides an effective means of modulating the structural integrity and gel strength, with increasing protein concentration (φ) promoting more pronounced solid-like behavior [49]. Furthermore, gelation behavior is also affected by the ionic strength of the medium. Electrolytes can alter protein conformation. In SPI systems, a moderate increase in ionic strength tends to expose hydrophobic regions, which improves affinity toward the oil phase and enhances OBC. This effect is not linear. When ionic strength exceeds a certain range, network stability decreases, mainly due to electrostatic shielding of charged groups [50].

Apart from concentration effects, the chemical nature of oleogelators is a primary factor controlling microstructure [51]. Wax-based systems usually form needle-like crystals that build rigid frameworks. Monoglycerides tend to assemble into hydrogen-bonded crystalline networks with adjustable morphology. Polysaccharides mainly contribute through chain entanglement and viscosity increase. Proteins behave differently, forming amphiphilic networks driven by interfacial adsorption and intermolecular interactions [52]. These differences are reflected in mechanical strength, thermal behavior, and oil retention.

Structure and performance can be further tuned using multi-component systems. Combining different oleogelators often leads to synergistic effects. In wax–monoglyceride systems, one component may provide heterogeneous nucleation sites while the other fills intercrystalline voids or modifies crystal habit, thereby increasing network connectivity. In sterol–lecithin or wax–lecithin systems, hydrogen bonding between polar head groups can alter molecular packing and generate denser supramolecular assemblies. Protein–polysaccharide combinations rely on electrostatic complexation, hydrogen bonding, hydrophobic association, and interfacial adsorption to connect oil droplets with the continuous biopolymer network [35,52]. In many cases, these hybrid systems show higher oil-binding capacity and stronger gels due to complementary structural roles. Protein–polysaccharide complexes, for instance, can reach oil-binding capacities above 97% under optimized conditions [53]. Wax-based blends can also reduce the required gelator amount while maintaining structural integrity [16]. A binary formulation should therefore be described as synergistic only when its response exceeds that of concentration-matched single-component controls prepared under the same thermal and shear history.

Soluble soybean polysaccharide (SSPS), a pectin-like acidic polysaccharide recovered from soybean-processing residues, is a potential alternative to gum arabic in emulsion-templated oleogels and bigels. SSPS can stabilize oil droplets through steric and electrostatic repulsion and can also reinforce the aqueous network of mixed gels [54]. Its use may reduce dependence on gum arabic, whose supply and composition can fluctuate; however, direct comparisons of SSPS- and gum-arabic-based oleogels in meat products remain limited and should be investigated.

Other components can also influence gel formation. Small amounts of emulsifiers, co-structurants, or minor phases such as water and salts are often introduced [2]. Emulsifiers and co-structurants regulate oleogel formation by altering interfacial tension, gelator wetting, nucleation, polymorphic transitions, and the connectivity of the continuous network. Lecithin may act as a crystal-habit modifier and can either weaken or reinforce glyceryl-stearate networks depending on its hydrogenation state and concentration [33]. Mono- and diglycerides may co-crystallize with waxes or hard fats, whereas water can create capillary bridges or serve as the dispersed phase in emulsion-templated oleogels. Hydrocolloids increase the viscosity of the aqueous phase and stabilize droplets before drying or solvent exchange. Ionic strength has a non-linear effect: moderate salt levels can enhance hydrophobic association and protein adsorption, whereas excessive electrostatic screening promotes aggregation and network collapse [49,50,53,55].

Oil refining status is an additional but underexamined variable. Crude or expeller-pressed oils contain phospholipids, free fatty acids, sterols, pigments, waxes, and oxidation products that change polarity and may serve as nucleation promoters or, conversely, interfere with ordered gelator crystallization. In the rice-bran-wax/corn-germ-oil system, refined oil produced a stronger gel than expeller-pressed oil [23], indicating that the removal of polar minor components improved wax-network development. This outcome should not be generalized, because the effect depends on the identity and concentration of the minor components and on the gelator mechanism.

At the molecular level, oleogelation involves a combination of non-covalent interactions, including van der Waals forces, hydrogen bonding, hydrophobic interactions, and electrostatic effects [56]. Which interaction dominates depends on the system. These interactions together define crystal morphology, network density, and rheological behavior. A higher storage modulus (G′) than loss modulus (G″) generally indicates a stronger solid-like structure, which is essential for mimicking traditional solid fats in food systems [57].

Overall, oleogel properties are determined by the combined effects of oleogelator type, concentration, molecular structure, and processing conditions. A rational formulation strategy that considers these factors can better control microstructure and improve functional performance, supporting the development of oleogels as practical fat substitutes.

### 2.3. Processing Conditions Governing Oleogel Formation

Processing conditions strongly influence oleogel formation by regulating crystallization kinetics, network assembly, and final macroscopic properties [2,9]. Among these variables, cooling rate, mechanical shear, and emerging physical technologies such as ultrasonication are particularly important, as they directly affect nucleation, crystal growth, and network organization (Table 2).

#### 2.3.1. Thermal History and Cooling Rate Effects

**Figure 2:**
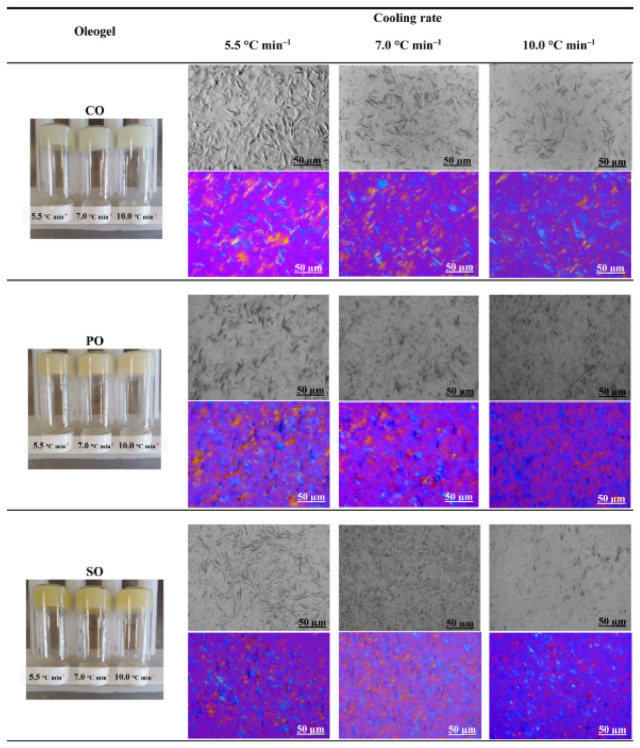
Test tube inversion test, physical appearance, bright-field light (top) and polarized light microscopy (bottom) images of canola oil (CO), peanut oil (PO), and sesame oil (SO) oleogels, prepared under different cooling rate conditions [11].

Cooling rate is a key factor governing oleogel structure development, as it determines heat transfer and crystallization pathways [58]. In general, rapid cooling promotes early nucleation and restricts crystal growth, resulting in a larger number of smaller crystals and a more compact network (Figure 2). This refined microstructure is often associated with higher gel hardness, improved oil-binding capacity, and enhanced viscoelastic stability.

Conversely, crystal growth is favored over nucleation under slow cooling conditions. This leads to fewer but larger crystalline domains and a less interconnected network [59], which typically weakens mechanical strength and structural integrity [58]. However, this trend is not universal. The oleogelator system and its self-assembly pathway play key roles in determining the influence of cooling rate. For wax-based systems, faster cooling generally strengthens gels due to finer crystal networks. In monoglyceride- and sorbitan-based systems, the outcome is more complex, as crystallization competes with lamellar phase formation [60,61]. These observations indicate that cooling rate alone cannot define final gel properties; its effect is tightly coupled with molecular structure.

#### 2.3.2. Ultrasonication as an Emerging Structuring Technology

**Figure 3:**
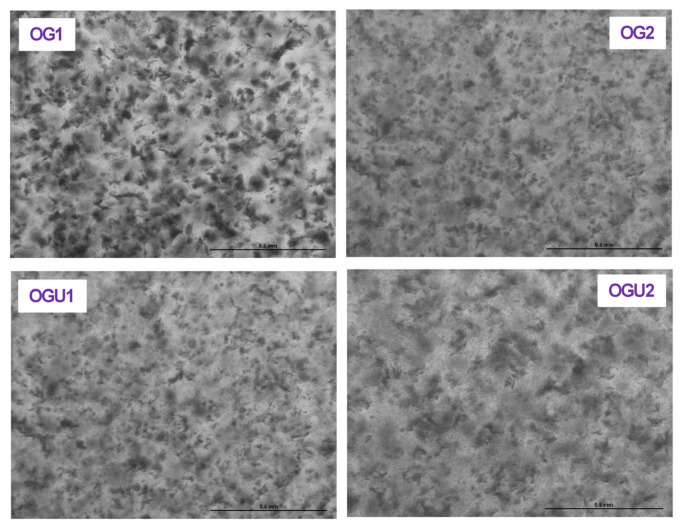
Microscopic observation of palm oil-based oleogels prepared with different concentrations of carnauba wax and the ultrasonication process (OG1, palm oil + 5% carnauba wax; OG2, palm oil + 10% carnauba wax; OGU1, palm oil + 5% carnauba wax + ultrasonicated; OGU2, palm oil + 10% + ultrasonicated). Reproduced from [40].

High-intensity ultrasound (HIU) has recently been introduced as an efficient and relatively green approach for tuning oleogel microstructure [62,63,64]. Acoustic cavitation generates localized temperature and pressure fluctuations, which improve oleogelator dispersion, enhance dissolution, and promote sonocrystallization [64]. As a result, crystal size decreases, nucleation density increases, and network uniformity improves (Figure 3).

These structural changes are generally reflected in improved oil-binding capacity, hardness and viscoelastic behavior, without altering chemical composition [62,65]. For instance, a 3 min ultrasound treatment significantly reduced crystal dimension and crystal occupancy (*p* < 0.05), while increasing hardness without noticeable changes in G′ or oil loss compared with untreated samples [66]. In addition, ultrasound can reduce oleogelator requirements, suppress phase separation, and improve sensory texture, making it attractive for clean-label fat structuring [64,65,67]. Beyond single-component systems, ultrasound may also strengthen interactions in multi-component oleogels, thereby improving structural stability. A detailed overview of ultrasound-assisted oleogelation can be found in recent reviews [68].

#### 2.3.3. Mechanical Shear and Mixing Effects

Mechanical shear plays a direct role in determining oleogel structure by influencing crystal dispersion, nucleation behavior, and network homogeneity. Moderate shear improves oleogelator distribution in the oil phase and promotes more uniform nucleation, leading to improved gel consistency and mechanical performance. Under these conditions, crystallization may also shift material behavior from brittle-like fracture toward more ductile deformation [69].

However, the effect is highly dependent on shear intensity. Excessive shear can disrupt early network formation by breaking growing crystal structures and weakening gelator–gelator and gelator–oil interactions, ultimately reducing gel strength [28]. In contrast, insufficient shear may lead to poor dispersion, phase separation, and heterogeneous microstructures, which negatively affect texture and oil-binding performance [70,71].

Taken together, processing variables do not act independently. Cooling rate, ultrasonication, and shear interact with each other, jointly controlling nucleation kinetics, crystal morphology, and network connectivity. These microstructural features ultimately determine macroscopic properties such as hardness, elasticity, oil retention, and sensory behavior. Therefore, integrated control of processing conditions is essential for rational oleogel design [69].

**Table 2:** Application of processing conditions to produce oleogels.

Processes	Oleogelators	Conditions	Oil Phases	Main Findings	References
Ultrasonication					
	Beeswax	Amplitude, 45.45%; time, 10.15 min	Rice bran oil	Ultrasonication significantly enhanced gel strength, hardness, and thermal stability without altering chemical integrity.	[12]
	Beeswax, candelilla wax, carnauba wax, and rice bran wax	Amplitude, 50%; time, 10 s; probe, 13 mm	Rapeseed oil	After sonication, beeswax, candelilla wax, and carnauba wax waxes significantly improved, with mostly physical properties evaluated. On the contrary, RBW showed a depletory effect of physical properties after sonication in the condition tested.	[62]
	Carnauba wax	Amplitude, 35.40%; time, 16.33 min	Soybean oil	The oil binding capacity of the oleogel increased from 91.4% to 98.7%, hardness increased from 52.56 to 290.22 N/mm^2^, and similarly, adhesiveness varied from −20.30 to −101.72 N/mm^2^. Ultrasonication strengthened the oleogels and made it more stable over a wider strain range than the control oleogel sample.	[65]
	Monoglycerides	Amplitude, 40%; time, 1 min; probe diameter, VS 70 T with 13 mm diameter	Olive oil	Ultrasonication accelerated the crystallization of the beta-polymorph of the monoglycerides, and resulted in shorter and thinner β-monoglyceride crystals. Ultrasonication induced the formation of stronger but lower-yield-stress oleogels.	[72]
	Fully hydrogenated mono- and di-glycerides	Amplitude, 25% or 50%; time, 15 or 30 s; frequency/power, report from source or NR	Soybean oil and avocado oil	High-intensity ultrasound (HIU) reduced crystal size and formed needle-like small crystals in all treated samples when compared to control, regardless of the oil source. HIU treatments increased the hardness of all samples in contrast to control.	[63]
	Rice bran wax, soybean protein isolate, and phosphatidylserine	Power, 0–600 W; time, 15 min; frequency, report from source or NR	Walnut oil	When the ultrasonic power was 450 W, the oil binding capacity reached 95.3%, which was the best compared with other groups. Ultrasonic treatment of appropriate power succeeded in making the soybean protein isolate-phosphatidyl-serine-walnut oil oleogels more evenly dispersed in the internal structure and denser in the external structure.	[64]
	Carnauba wax	Frequency, 25 kHz; time, 10 min; power, report from source or NR	Palm oil	The combined use of ultrasonication and a higher carnauba wax concentration enhanced the properties and storage stability of palm oil-based oleogel.	[40]
	Ethyl cellulose	Frequency, 20 kHz; power, 240 W; tip diameter, 20 mm; duty cycle, 10%; temperature, 40 °C	Medium-chain triglyceride liquid oil	Compared with oleogels prepared by conventional stirring, ultrasound-assisted medium-chain triglyceride oleogel produced using a 10% duty cycle at 20 kHz exhibited a higher elastic modulus, lower oil loss after 30 days of storage, and superior hardness and stickiness throughout the storage period.	[73]
	Cellulose particles and sorbitan fatty acid esters	Frequency, 40 kHz; power, 480 W; time, 2 min; temperature, 25 ± 1 °C; probe diameter, 0.6 mm	Medium-chain triglycerides	Ultrasonic treatment enabled a combination of 6% (*w*/*w*) cellulose particles and 9% (*w*/*w*) sorbitan fatty acid esters to form a robust network capable of effectively entrapping the oil phase.	[74]
	Monoglycerides, fully hydrogenated rapeseed oil, and lecithin	Frequency, 20 kHz; power, 50 ± 2 W (1 W/mL); amplitude, 50%; time, 10 s	Rapeseed oil	The improvements in physical properties, including hardness, elastic modulus, and oil loss, following sonication were attributed to HIU-induced secondary crystallization of FHRO.	[67]
	Candelilla wax	Frequency, 20 kHz; power, 95 W; probe diameter, 6.36 mm; amplitude, 10%; 2 s ON/2 s OFF; total sonication time, 10 s	Peanut oil, pine nut oil, walnut oil	HIU significantly improved the crystal strength and physical properties of peanut, pine nut and walnut oleogels. All HIU-treated oleogels showed more crystals under polarized light microscopy. HIU improved G′ values but reduced oil loss of peanut, pine nut and walnut oleogels.	[75]
	Monoglycerides (MG) and high-melting-point triacylglycerols (HF)	Frequency, 20 kHz; power, 56 W; amplitude, 50%; macro-tip diameter, 12.7 mm; treatments of 10 s, 30 s, or three 10 s pulses with 10 s OFF intervals	Rapeseed oil	In contrast to the blends containing both MG and HF, sonication adversely affected the properties of MG6:HF0 (6% monoglycerides and 0% high-melting-point triacylglycerols). By comparison, sonication exerted a beneficial effect on the MG/HF blends, increasing hardness by at least threefold and oil binding capacity by approximately 20%. Notably, these improvements were evident after only 10 s of sonication.	[17]
	Sorghum bran waxes	Power, 110 W; frequency, 40 kHz; time, 5 min	Fish oil	Fast cooling and ultrasonic treatment reduced the crystal size. Fast cooling and ultrasound favored the oil-gelling capacity and reduced oil loss.	[76]
	Monoglycerides	Frequency, 20 kHz; power, 96 W; 3 pulses, 10 s on/5 s off	High-oleic sunflower oil	HIU induced remarkable alterations in monoglyceride crystallization behavior, shortening crystal length and generating a stronger, more elastic network with elevated oil binding capacity. HIU increased the adhesiveness of all samples while exerting no significant influence on cohesiveness.	[77]
	Propolis wax	Frequency, 20 kHz; probe diameter, 13 mm; power, 100–300 W; times, 30–120 s	Olive oil	HIU-induced nucleation by creating small crystals led to formation of a strong network with high oil binding capacity.	[78]
	Monoglycerides	Probe diameter, 3.2 mm; frequency, 20 kHz, power, 96 W; time, 30 s in pulse mode: 10 s on/5 s off; cooling rate, 0.1 and 10 °C/min	High-oleic sunflower oil	Compared with oleogels cooled at 10 °C/min, those prepared under a slow cooling rate exhibited a lower storage modulus after 24 h. In addition, increasing the MG concentration and cooling rate, as well as applying HIU, enhanced the network oil binding capacity.	[79]
	Candelilla wax, monoacylglycerol, and hard fat	Tip diameter, 3.2 mm; vibration amplitude, 216 µm; time, 3 min; frequency/power, report from source or NR	High-oleic sunflower oil	When HIU was applied to the oleogel for 3 min using a 3.2 mm-diameter tip at an amplitude of vibration of 216 μm, a reduction in crystal size and crystal area was observed with an increase in hardness and no change in storage modulus nor in oil loss compared to the nonsonicated oleogel. Other sonication conditions (lower power levels, shorter durations, and bigger tips) tested in this study reduced the hardness and elasticity of the sample and increased oil loss.	[66]
Cooling rate					
	Beeswax	5.5, 7.0, and 10.0 °C/min	Canola oil, peanut oil, and sesame oil	Higher cooling rates raised hardness and adhesiveness for stronger deformation resistance, while reducing springiness and cohesiveness, which improved spreadability suitable for solid fat applications.	[11]
	Beeswax and stearic acid	0.5, 1.5, and 5.0 °C/min	Rice bran oil blend and sesame oil	The gel network strength was markedly affected by the cooling rate. Compared with rapid cooling, a cooling rate of 0.5 °C/min produced oleogels with higher oil-binding capacity and a more robust gel structure. In contrast, the thermal properties and molecular interactions of the oleogels remained unchanged regardless of the cooling rate.	[80]
	Candelilla wax	0.5, 2.0, and 5.0 °C/min	Rapeseed oil	Fast cooling rate resulted in smaller crystals, which facilitated the formation of oleofoams with higher foamability.	[81]
	Ceramide	4 °C for fast cooling and 30 °C for slow cooling for 24 h before further analysis	Sunflower oil	The slower cooling rate resulted in larger crystals adsorbed at the droplet surface.	[82]
	Sunflower wax, beeswax, and their hydrolyzed variants	0.1, 3.0, and 20.0 °C/min	Refined canola oil	Hardness increases with increasing cooling rate.	[83]
	Glycerol monostearate	Cooling temperature (25 °C and 4 °C)	Sunflower oil	Increasing the glycerol monostearate concentration and decreasing the cooling temperature altered the self-assembled structure of glycerol monostearate oleogels, leading to the formation of smaller crystals and a denser network, which consequently enhanced the hardness, oil-binding capacity, and viscoelastic properties of the oleogels.	[84]
	Sunflower wax, rice bran wax, candelilla wax, or beeswax	0.05 and 10.0 °C/min	Sunflower and canola oil	Highly ordered wax crystal structures are more likely to develop in oils lacking polar components under slow cooling conditions.	[85]
	γ-Oryzanol and β-sitosterol	1.0 and 7.0 °C/min	High-oleic sunflower oil	Different cooling rates influence the texture of oleogels.	[86]
	Lecithin and ceramide	2.0, 5.0, 10.0, 15.0, and 20.0 °C/min	Sunflower oil	The lower cooling rate resulted in ceramide/lecithin oleogel with a higher storage modulus. The lower cooling rate also resulted in ceramide/lecithin oleogel with a larger crystal size. The cooling rate influenced the crystallization kinetics of ceramide/lecithin oleogel.	[59]
	Beeswax	1.0, 5.0, and 10.0 °C/min	Camellia oil, corn oil, sunflower oil, and linseed oil	Contracted crystals gathered significantly at a slow cooling rate.	[42]
	Sorghum bran wax, sorghum DDGS wax, and sorghum kernel wax	4.1 ± 0.2, 7.8 ± 0.2, and 12.8 ± 1.2 °C/min	Fish oil	Fast cooling and ultrasonic treatment reduced the crystal size. Fast cooling and ultrasound favored the oil-gelling capacity and reduced oil loss.	[76]
	Wax ester	0.8, 5.0, and 10.0 °C/min	Medium-chain triglycerides oil	Higher cooling rates accelerate nucleation and shorten the induction period. In contrast, the results indicate that slower cooling rates tend to promote the formation of more regular crystal habits.	[87]
	Monoglycerides	0.1 and 10.0 °C/min	High-oleic sunflower oil	A slower cooling rate led to a lower storage modulus after 24 h than that achieved at 10 °C/min. Moreover, the network oil binding capacity increased with increasing cooling rate.	[79]
Shear rate					
	Beeswax and candelilla wax	0, 10, 130 s^−1^	Canola oil	Applying high shear rates during crystallization may weaken the mechanical strength of oleogels. Moreover, dynamic crystallization conditions can modify the mesoscopic structure of oleogels, leading to a more plastic and softer system resembling commercial margarine.	[69]
	Beeswax	0, 10, 50, 90, and 130 s^−1^	Canola oil	Sheared oleogels display plastic-like behavior, lower linear elastic moduli, and a higher perfect plastic dissipation ratio than oleogels cooled under quiescent conditions, which displayed stiff, brittle-like characteristics. In addition, these oleogels displayed a microstructure with smaller crystals than oleogels cooled under quiescent conditions.	[88]

### 2.4. Critical Comparison and Structure–Function Design Rules

Across the literature, oleogel performance cannot be evaluated solely based on gel strength, as different structuring strategies inherently involve a balance among multiple functional properties [89]. Low-molecular-weight crystalline oleogelators, such as waxes and monoglycerides, are highly efficient in immobilizing liquid oils at relatively low concentrations. However, the resulting crystalline networks are often rigid and vulnerable to changes in thermal history, with storage-induced polymorphic transitions potentially affecting long-term stability. In contrast, ethyl cellulose provides robust polymeric networks with excellent thermal resistance, making it suitable for heat-processing applications. Nevertheless, its practical use is limited by the requirement for high-temperature dissolution and the potential formation of overly firm textures or undesirable oral sensations. Protein- and polysaccharide-based indirect structuring approaches offer advantages in terms of clean-label acceptability and mild processing conditions, yet they typically require additional steps such as emulsion formation, drying, or solvent exchange, and their structural stability can be strongly influenced by residual moisture [90].

Multi-component systems provide the greatest design flexibility but also the greatest uncertainty [2,91]. The improved performance observed in a binary or hybrid formulation should not be directly attributed to synergistic interactions unless it is validated against single-component systems containing equivalent amounts of structuring agents and processed under comparable thermal and mechanical conditions. Otherwise, enhanced hardness or OBC may simply reflect an increased total structurant concentration rather than cooperative assembly between different components. In addition, interactions among multiple structuring agents are not always beneficial; incompatibility in crystallization behavior, competition for interfacial regions, or interference with network formation may weaken the resulting structure.

Therefore, the design criteria for oleogels should be closely linked to their intended application rather than relying on a universal indicator of structural performance. For bakery products, desirable oleogels should provide sufficient plasticity, air incorporation capacity, and appropriate melting behavior. In plant-based meat systems, maintaining juiciness, thermal stability, and fat-like phase transitions is more critical [5], whereas frying applications prioritize oxidative resistance and structural integrity during repeated heating [48]. Similarly, oleogels used as bioactive delivery systems require precise control over lipid digestion and compound release. These diverse requirements highlight that the optimal oleogel structure is application-dependent, and successful formulation should be achieved by balancing the intrinsic assembly mechanism of the structuring system with the processing environment and final product characteristics.

Further progress in oleogel research also depends on improving the consistency of experimental reporting. Besides basic formulation information, future studies should provide detailed descriptions of oil composition and minor constituents, gelator purity, thermal and shear processing history, storage conditions, rheological parameters (G′, G″, and yield stress), OBC measurement protocols, crystal morphology and polymorphic forms, oxidative stability, and digestion behavior. Such standardized characterization would facilitate more reliable comparisons across studies and support the development of quantitative structure–property relationships, moving oleogel design beyond application-specific trial-and-error optimization.

## 3. Practical Applications of Structurally Modulated Oleogels

**Figure 4:**
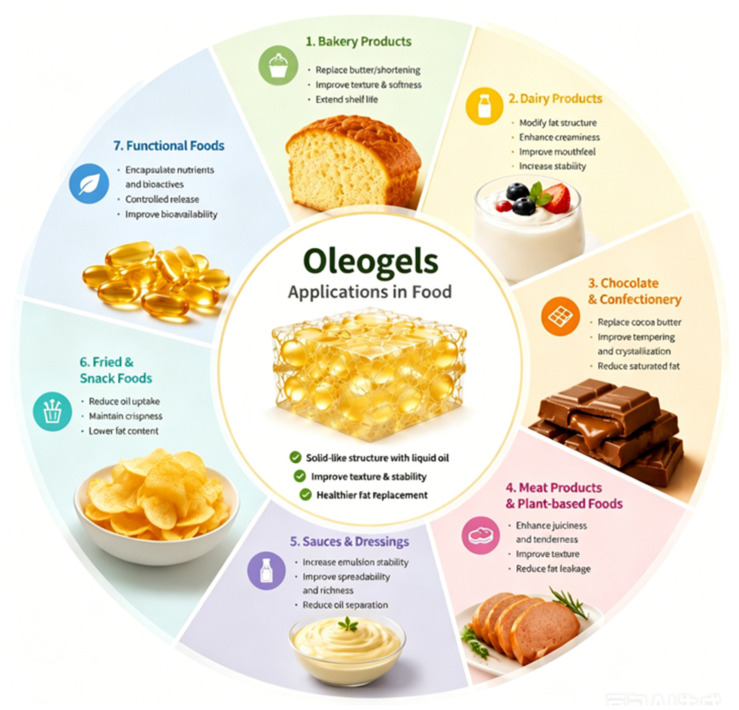
Application of oleogels in food product.

Recent progress regarding lipid-structured oleogels applied within bakery, dairy, meat, spreads and confectionery sectors, alongside other relevant fields, is outlined in Figure 4, with comprehensive data listed in Table 3. Oleogel applications have been extensively reported in a wide range of food systems, including bakery products [56,92], meat products [93,94], dairy products [95], and confectionery and chocolate [96] formulations; therefore, these areas are not discussed in detail here. Instead, this section focuses on recent advances in the application of oleogels in plant-based meat products, fried foods, and functional foods where they have shown particular promise as structured lipid systems for fat replacement and quality improvement.

### 3.1. Plant-Based Foods

The growing demand for sustainable, healthier, and more ethical protein sources has accelerated the development of plant-based meat analogs (PBMAs) [97]. While substantial progress has been made in reproducing muscle-like fibrous structures through protein modification, the design of lipid components remains comparatively underexplored. This is a critical gap, as fat plays a central role in determining juiciness, texture, flavor release, and overall palatability [98]. As a result, structuring edible oils into solid-like fat mimetics has become an important strategy for improving the sensory quality of PBMAs [99].

Among available approaches, oleogelation has attracted increasing attention. It enables the conversion of liquid oils into semi-solid materials using food-grade structuring agents, offering a trans-fat-free and low-saturated-fat alternative to conventional fats [5,99]. In PBMA systems, oleogels have been shown to improve thermal stability, texture, and overall eating quality. For example, canola oil oleogels structured with candelilla, carnauba, or beeswax can effectively replace coconut oil while enhancing product stability [5]. Similarly, incorporation of 20% candelilla wax oleogel has been reported to reduce thawing and cooking losses and improve sensory acceptance in plant-based patties [97].

The functionality of oleogels in these systems depends strongly on both oil composition and gelator type. Ethyl cellulose remains one of the most widely studied oleogelators due to its strong thermal stability and network-forming ability. In addition, enzymatically modified oils containing partial glycerides have attracted attention, as they can alter crystallization behavior and texture development. For instance, Soleimanian, Ghazani and Marangoni [100] reported that ethyl cellulose-based oleogels prepared from glycerolysis-modified oils reduced oil leakage during cooking, thereby improving the juiciness and texture of PBMAs.

Structurally, oleogels can partially mimic the functional behavior of animal fat. Texture profile analysis has shown that refrigerated oleogels can reproduce key attributes of pork and beef fat, including hardness and chewiness [97]. In meat emulsions and frankfurters, oleogel substitution often yields hardness values comparable to animal fat-based formulations. Thermal behavior further indicates reduced fat and moisture loss during cooking, and in some cases, a fat-like melting profile similar to bovine adipose tissue. These effects contribute to improved product stability and sensory quality.

Despite these advances, several limitations remain. Current oleogel systems rarely replicate the full complexity of animal adipose tissue, particularly under real processing conditions [5]. In addition, inconsistencies between instrumental texture analysis and sensory perception are still not well resolved. Other challenges include the brittleness of certain oleogelators such as EC, structural instability during thermal processing, and limited availability of low-cost, clean-label alternatives.

Overall, oleogels provide a flexible platform for tailoring lipid functionality in PBMAs. Future research should focus on clarifying structure–sensory relationships, improving processing robustness, and developing more food-friendly oleogelators that better mimic the multi-scale behavior of animal fat.

### 3.2. Fried and Snack Foods

Deep-fried foods are widely valued for their crisp texture and flavor intensity [101]. However, conventional frying oils are associated with excessive oil uptake, oxidative degradation, and formation of undesirable by-products during repeated use [102]. These issues have driven interest in healthier frying media. Oleogels have emerged as promising frying mediums due to their ability to structure liquid oils into semi-solid systems with improved thermal and oxidative stability [103].

**Figure 5:**
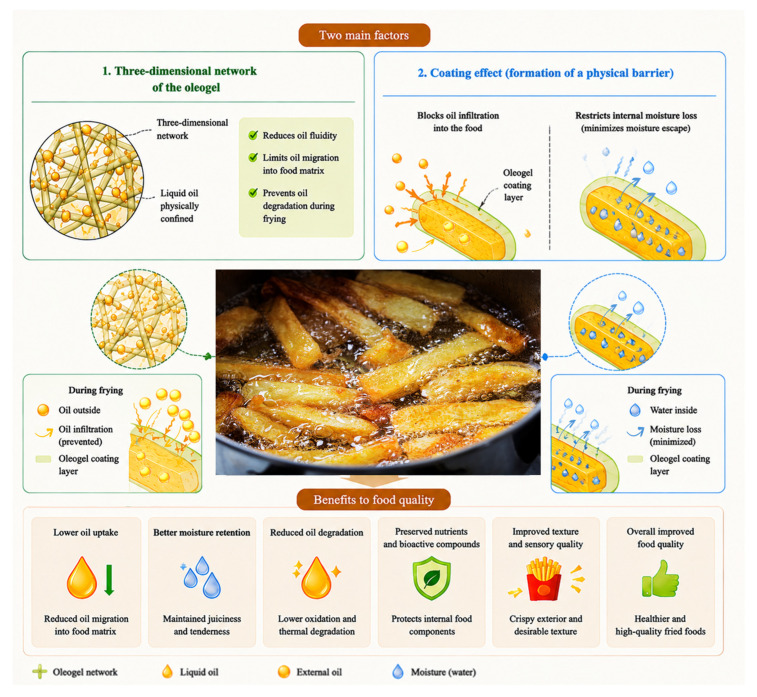
The mechanism of oleogel frying in improving food quality. Adapted from [48].

Compared with conventional oils, oleogel-based frying systems can reduce oil absorption by approximately 20–50% [48], while improving moisture retention, texture, and color uniformity [104]. Such improvements can be largely ascribed to the physical network of oleogels, which restrains liquid oil and hinders its migration into food matrices. In addition, oleogels can form a transient coating layer on food surfaces during frying [48], limiting oil penetration and moisture loss. Furthermore, volatile components, particularly ketones and esters, were significantly increased in rice cakes fried with oleogel, further improving sensory quality and acceptability by frying with oleogel [105]. Together, these effects contribute to improved product quality and reduced fat uptake [48], as illustrated in Figure 5.

Several studies have confirmed these benefits. Doughnuts fried in sunflower oil–soybean wax oleogels showed reduced oil absorption and improved appearance compared with conventional oil frying [106]. Similarly, another study reported that oleogel-fried potato chips absorbed approximately 23% less oil than conventionally fried chips while maintaining comparable sensory attributes and fatty acid composition [107]. Comparable reductions in oil uptake have also been observed in fried meat and vegetable products, highlighting the broad applicability of oleogels across different food matrices [48].

However, most studies remain limited to laboratory-scale conditions. Long-term frying stability, cost-effectiveness, and consumer acceptance are still insufficiently investigated. In addition, the relationship between oleogel microstructure and frying performance remains unclear. Future work should therefore focus on optimizing formulations for repeated frying cycles and evaluating industrial scalability. Additional information regarding oleogel applications in frying systems can be found in recent reviews [108].

**Table 3:** Application of oleogels in food systems.

Food Product	Oleogelators	Oil Phase	Main Findings	References
Bakery Products				
Gluten-free bread	Pectin and egg yolk low-density lipoprotein	Soybean oil	The oleogel-based bread exhibited greater specific volume and superior softness and elasticity.	[109]
Cookies	Beeswax	Rice bran oil	Sensory evaluation demonstrated that the developed oleogels were comparable to butter-based controls in appearance, texture, flavor, and overall acceptability, indicating their potential as a viable fat replacer in bakery products without compromising consumer perception.	[12]
Croissants	Glycerol monostearate, lecithin, and sucrose ester	Sunflower oil	Butter replacement (50%) reduced hardness, and elevated volume by 2.48 mL/g.	[110]
Yellow cake	Rice bran wax or carnauba wax	Corn oil	Oleogel-based cakes matched shortening in sensory acceptance.	[111]
Cake	Ethyl cellulose, glycerol monostearate, and whey protein isolates	Triacylglycerol or diacylglycerol	Whey protein isolate oleogel cakes exhibit favorable organoleptic qualities.	[112]
Burgers	Pig skin	Olive oil	A healthier low-fat burger with high sensory acceptability among panelists was successfully developed without any additives.	[113]
Bread	Insoluble soybean fiber	Soybean, sunflower, corn, peanut, and flaxseed oils	These oleogels have similar organoleptic properties to commercial bread and have great potential to partially replace butter in bakery applications.	[114]
Gluten-free rice-flour cakes	Hydroxypropyl methylcellulose and chia seed mucilage powder	Chia seed oil and canola oil	The formulation containing 50% oleogel without curcumin achieved the best balance of technological, nutritional, and sensory qualities.	[115]
Bread	Whey protein isolate and *l*-lysine	Soybean oil	The application of this oleogel in bread making showed that a 50% replacement of butter achieved the most ideal texture.	[116]
Dairy Products				
Mozzarella cheese analog	Yellow beeswax	Soybean oil	Replacement of shortening with beeswax oleogels resulted in cheese samples with harder and more cohesive textures.	[117]
Processed cheese	Beeswax	Corn oil and mustard oil	The processed cheese with oleogel exhibited more significant antioxidant activity (63.30%) than the control cheese sample (31.86%).	[118]
Filling cream	Carnauba wax	Sunflower oil, camelina oil, and a combination of oils	The formulated creams with substitution levels of up to 50% exhibited acceptable peroxide values and oil retention characteristics. Increasing the replacement level consequently reduced hardness and adhesiveness, which contributed to a decrease in specific gravity	[119]
Ice cream	Beeswax, carnauba wax, and candelilla wax	Canola oil	Wax oleogels are a technological and healthy viable alternative to replace milk fat in ice cream.	[120]
Non-diary whipped creams	Glycerol monostearate and glycerol monolaurate	Sunflower oil	Increasing the oleogel ratio enhanced the fat crystal network with a higher density. Interfacial, rheological, and whipping properties improved at higher oleogel ratios.	[121]
Vanilla ice cream	Chitosan	Soybean oil	The fat incorporation method had a greater influence on the technological properties of oleogel ice cream than on milk ice cream.	[122]
Fat-reduced ice creams	Monoglyceride	Refined soybean oil	Ice cream with 50% oleogel possessed an appropriate partial coalescence degree, and the complete network structure endowed ideal qualities and the generation of perceived creaminess	[123]
Aerosol whipped cream	Beeswax and carnauba wax	Soybean oil	Beeswax:carnauba wax (8.5:1.5) achieved the best overrun, stability, and gas retention	[124]
Vegan cheese	γ-Oryzanol and β-sitosterol	Sunflower oil and extra virgin coconut oil	Oleogel-based vegan cheese properties were highly tunable, with hardness and adhesiveness levels which could be in excess or below that of the control samples.	[125]
Aerosol whipped cream	Beeswax, rice bran wax, and carnauba wax	Soybean oil	Whipped cream with 6% beeswax showed significantly improved overrun and stability, and its saturated fatty acid concentration was 4.4-fold lower than that of dairy milk fat whipped cream.	[126]
Chocolate and confectionery				
Chocolate cream fillings	Sunflower wax or glycerol monostearate	Corn oil or sesame oil	Cream fillings formulated with 6% glycerol monostearate-based oleogels (especially the corn-oil oleogel containing 6% glycerol monostearate) exhibited favorable sensory acceptability, softer textures, and greater thermal stability compared to the traditional shortening control.	[127]
Confectionary cream	Carnauba wax and beeswax	High-oleic sunflower oil	Naïve consumers did not consider softer texture as knock-out criteria, and overall acceptance of the creams with oleogels was not significantly different from the classical ones.	[128]
Chocolate spread	Diacetyl tartaric acid ester of mono(di)glycerides/beeswax	Soybean oil	A 20% lipid replacement using 5% mono(di)glycerides/2% beeswax not only achieved superior sensory evaluation but also endowed the micro-aeration chocolate spread with exceptional 3D printability.	[129]
Chocolate spreads	Soy protein concentrate and carrageenan	Red palm oil	The potential of biphasic gelation of red palm oil is an effective approach to structure red palm oil for use as a cocoa butter replacer in chocolate spreads.	[130]
Meat products and plant-based foods				
Low-saturated-fat plant-based meat analogs	Candelilla wax, carnauba wax, and beeswax	Canola oil	Wax-based oleogels were promising solid fat alternatives for developing plant-based meat analogs with enhanced cooking performance and healthier fatty acid composition.	[5]
Chicken breast and pork hind leg	β-Cyclodextrin, xanthan gum, and monoglyceride	Refined camellia oil	Compared with conventional NaCl dry curing, oleogel-mediated curing using a formulation containing 15% NaCl resulted in chicken breast and pork hind leg products with acceptable salt content, better visual quality, increased tenderness and water-holding capacity, while reducing hardness, cooking loss, and oxidation degree.	[26]
Beef burgers	Carnauba wax	Grapeseed oil	Oleogel substitution reduced hardness by approximately 40% while preserving springiness and cohesiveness.	[131]
Deep-fried chicken nuggets	β-Sitosterol/γ-oryzanol	Rice bran oil	β-Sitosterol/γ-oryzanol oleogels serve as an effective delivery system for curcumin, simultaneously reducing fat, oxidative degradation, and toxic compounds while preserving sensory quality in fried chicken products.	[132]
Emulsified sausage	Flaxseed gum and arabic gum	Soybean oil	The prepared oleogels, serving as fat substitutes, enhanced the textural characteristics and nutritional quality of emulsified sausages. As the oleogel substitution level increased, the hardness and chewiness of emulsified sausages increased, whereas cooking loss was reduced.	[133]
Meat sausage	Almond flour and xanthan gum	Sesame oil	The addition of sesame oil oleogel and almond flour proved to be a good alternative for replacing fat in meat products.	[134]
Plant-based meat patties	Rice bran wax, candelilla wax, carnauba wax, and beeswax	Canola oil	Oleogels with 20% candelilla wax could be an effective replacement of plant oils in plant-based meat patties.	[97]
Hybrid meat patties	Hydroxypropyl methylcellulose (HPMC)	Sunflower oil	The greatest reductions in lightness, hardness, and chewiness were observed in patties containing the HPMC/xanthan-gum oleogel.	[135]
Luncheon meat	Soy protein hydrolysate-nano soy fiber	Soybean oil	Oleogel improved the fatty acid nutritional composition of luncheon meat.	[136]
Beef burgers	Beeswax	Moringa oil	Texture analysis revealed oleogel as the hardest.	[137]
Fried and snack foods				
Proso millet rice cakes	Carnauba wax	Soybean oil	Oleogel frying improved both oil stability and fried food quality.	[105]
Sliced potatoes	Candelilla wax	Extra virgin olive oil	The oleogel demonstrated better stability than extra virgin olive oil during frying. Meanwhile, potato slices fried in extra virgin olive oil had an oil uptake rate of 14.51%, which markedly decreased to 10.29% (*p* < 0.05) when the oleogel was applied as the frying medium.	[138]
Fish filets	Rice bran wax	Canola oil	Oleogels can be utilized as alternative frying media for fish, delivering products with reduced calorie content and enhanced aromatic quality.	[139]
Potato chips	Beeswax, carnauba wax, candelilla wax, and rice bran wax	Soybean oil	All oleogels inhibited the formation of oxidation products (hydroperoxides, aldehydes and ketones) during deep-fat frying.	[140]
French fries	Sunflower wax and monoacylglycerols	Rapeseed oil	Lower total fat content was detected in French fries fried in MG oleogel (9.2 g/100 g); in contrast, fries prepared with rapeseed oil (13.2 g/100 g) and SFW oleogel (12.1 g/100 g) showed no significant difference.	[141]
Puffed potato starch chips	Rice bran wax and beeswax	Rapeseed oil	Rapeseed oil-based rice bran wax oleogel is preferable for use as a frying medium and shows great potential for future utilization.	[142]
Doughnut	Soybean wax	Sunflower oil	Sunflower oil–soybean wax oleogel lowered doughnut oil uptake by ~37.8% and optimized color parameters; despite elevated frying viscosity, reduced oxidation indicators demonstrated its enhanced stability for frying	[106]
Fried potato chips	Beeswax	Sunflower oil	Beeswax can be considered a natural preservative that improves the shelf life of fried potato chips as well as the frying stability of sunflower oil	[143]
Coated chicken	Carnauba wax	Sunflower oil	Sunflower oil-based oleogels containing 1.5% or higher carnauba wax, which feature lower saturated fat levels, can serve as frying media to enhance the quality of coated deep-fried chicken products	[144]
Potato chips	Glyceryl monostearate and soy lecithin	Sunflower oil	When incorporated into potato chips, this system improved saltiness perception, thus providing a promising route toward flavorful low-sodium food development.	[145]
Onion rings	Polyglycerol stearate	Sunflower oil	No negative effects of oleogel usage on the L* value, aroma, crispness/texture, and overall acceptability scores for the onion ring samples fried in the oleogels	[146]
Potato strips	Beeswax	Sunflower oil	Overall acceptability of potatoes fried in sunflower oil–beeswax oleogels at 8% organogelator concentration is the highest (8.50) among all. The prepared oleogels are found to be a quite promising frying medium in this study	[147]

### 3.3. Functional Foods

Oleogels have also gained attention as delivery systems in functional foods, particularly for lipophilic bioactive compounds. By immobilizing oils within a structured network, they provide both fat replacement and a protective microenvironment that enhances stability and bioavailability [148].

Lipophilic compounds such as carotenoids, curcumin, resveratrol, coenzyme Q10, and omega-3 fatty acids (Table 4) benefit significantly from oleogel encapsulation [149]. These compounds are typically unstable and poorly soluble in aqueous environments. Incorporation into oleogels improves dispersion in lipid phases, reduces oxidative degradation, and facilitates micelle formation during digestion, thereby enhancing absorption. At the same time, the gel network acts as a barrier against oxygen, light, and heat.

Importantly, oleogel structure plays a key role in digestion behavior. Stronger networks or higher-molecular-weight oleogelators generally slow lipid hydrolysis, leading to more sustained release profiles. Recent studies have shown that ethyl cellulose- and wax-based oleogels can reduce lipid digestion rates and modulate β-carotene bioaccessibility [150]. Similarly, MG-crystal-based systems can entrap curcumin and slow its release during in vitro digestion [151]. Hybrid systems such as bigels further extend functionality by enabling co-delivery of hydrophilic and lipophilic compounds [14]. For instance, increasing oleogel content in MG–beeswax/gellan gum bigels has been shown to slow lycopene release during simulated digestion [152].

Overall, oleogels provide a versatile platform that integrates fat replacement with controlled delivery of bioactive compounds. Their structural tunability and digestive responsiveness make them promising candidates for functional food design aimed at improving nutritional outcomes and managing diet-related diseases. More information can be available in published papers [149,153].

**Table 4:** Overview of recent developments in the delivery of bioactive oleogels.

Bioactive Compounds	Health Benefits	Oleogelators	Oil Phases	Main Findings	References
*Lactiplantibacillus plantarum*	Antioxidant activity	Soy hull polysaccharides, soy protein isolate, soy lecithin, and stearic acid	Soybean oil	Bigels enhanced probiotic survival in simulated digestion vs. free cells.	[154]
Astaxanthin	Antioxidant and inflammatory activity	Soy protein and arabinoxylan	*Acer truncatum* seed oil	Oleogels support sustained release in the stomach and targeted delivery of astaxanthin to the intestine, yielding a peak bioaccessibility of 88.84% and notably improving the in vitro bioavailability of astaxanthin.	[91]
Resveratrol	Anti-inflammatory, antioxidant, antibacterial, anti-pulmonary fibrosis and cardiovascular protection activity	Ovalbumin-proanthocyanidin-sodium alginate	Sunflower oil	The novel ovalbumin-proanthocyanidin-sodium alginate conjugate-based oleogel improved the lipolysis rate and bioaccessibility of resveratrol.	[25]
Blueberry derived resveratrol	Antioxidant activity	Lecithin and sitosterol	Soybean, peanut, and hemp oil	Hemp oil oleogels demonstrated superior delivery efficiency and chemical stability for resveratrol (*p* < 0.05).	[155]
Lutein	Antioxidant, anti-inflammatory, anti-cancer, and immune-modulating properties	Glycerol monostearate	Soybean oil	Supramolecular oleogels with denser crystal networks achieved improved lutein bioaccessibility when used for lutein encapsulation.	[156]
β-Carotene	Antioxidant activity	A sterol-mixture or refined white beeswax	High-oleic sunflower oil	The dynamic friction coefficient of sterol-based oleogels continuously declined, implying that this system may facilitate digestion and thereby improve β-carotene bioaccessibility.	[157]
Curcumin	Antioxidant activity	Tea polysaccharide conjugate and xanthan gum	Medium-chain triglyceride	Curcumin encapsulated in oleogel improved antioxidant activity and bioavailability.	[158]
Tea polyphenols and curcumin	Antioxidant activity	Glyceryl monostearate	Soybean oil	Tea polyphenols and curcumin showed a certain synergistic antioxidant effect in oleogels.	[159]
Fish oil, β-carotene, and β-sitosterol	Antioxidant activity	Beeswax and stearic acid	Rice bran oil and sesame oil	β-Carotene enhanced the oxidative stability of oleogels during storage.	[160]
Curcumin	Antioxidant activity	Lecithin, glycerol monostearate, and phytosterols	Coconut oil	Increased firmness of PS-liquid coconut oil oleogels benefits the retention and chemical stability of loaded curcumin.	[161]

## 4. Future Perspectives

### 4.1. Next-Generation Structuring Agents

The development of novel structuring agents is essential for overcoming the limitations of current edible oleogels and expanding their industrial applications. Although existing oleogelators can effectively structure liquid oils, many require high concentrations and exhibit limited versatility, making it difficult to balance functionality, sensory quality, cost, and clean-label requirements. Therefore, next-generation oleogelators should prioritize renewable feedstocks, efficient network formation at low concentrations, sensory compatibility, and scalable processing. Marine biopolymers, microbial fermentation products, and underutilized plant feedstocks possess diverse molecular architectures [162]. Representative materials, including alginate, κ-carrageenan, chitosan, xanthan gum, bacterial cellulose, pectins, arabinoxylans, and cellulose nanofibrils, can form ionic, helical, or interfacial networks, thereby improving oil retention, rheological properties, and sustainability. Beyond raw material exploration, rational design of multifunctional oleogelators is equally important. Synergistic combinations of proteins, polysaccharides, and plant waxes can generate functional advantages unavailable from single-component gelators [163], while biotechnology and molecular modification enable precise regulation of molecular self-assembly and crystalline network formation, improving structuring efficiency and processing adaptability [162]. Future studies should balance functionality, safety, cost, and large-scale manufacturability while clarifying the relationships between molecular design, hierarchical gel structures, and final product properties to accelerate industrial applications.

### 4.2. Hierarchical Multi-Scale Structural Regulation

In-depth insights into hierarchical structural control are vital for targeted edible oleogel design. Despite extensive progress in tuning oleogel traits via formula and processing adjustments, the causal chain between molecular arrangement and bulk functional performance is not fully clarified. Subsequent research thus needs to build a unified multi-scale theoretical framework bridging molecular interactions, crystal assembly, network construction and end-product characteristics [51]. On the molecular scale, intermolecular forces such as hydrogen bonds and hydrophobic interactions dominate oleogelator self-assembly and lipid binding, which lay the foundation for crystallization; thorough analysis of these forces enables customized screening of high-efficiency gelators. At the mesoscale, crystal shape, crystal form and aggregates decide network integrity and stability, so more work is required to reveal how cooling rate, shear force and thermal cycles alter nucleation and crystal growth to manipulate oil retention and network compactness [110]. Macroscopically, mechanical, thermal and digestive behaviors stem from multi-level stacked structures. Quantified structure–property correlations are necessary to forecast texture, oxidation resistance and lipid digestion, while interactions between oleogels and food ingredients like proteins and emulsifiers also merit exploration.

Overall, breakthroughs in oleogel technology rely on cross-scale research. Unified structure–function models will support precise development of new oleogels and speed up their industrial food application.

### 4.3. High-Order Structural Modulation: Engineering Stimuli-Responsive “Smart” Oleogels

Stimuli-responsive oleogels have emerged as a promising research hotspot that extends the limited function of traditional oleogels, which merely serve as fat substitutes [164,165]. By introducing responsive molecular interactions into gel networks, these oleogels can achieve controllable structural changes under external stimuli, thereby effectively regulating food texture, stability, digestion properties and bioactive compound release. For instance, a shear-responsive oleogel with improved lubrication in oral conditions was developed in a previous study and provided a comprehensive framework bridging molecular-level design principles with macroscopic lubrication performance, and offered solutions for sustainable fat replacement and personalized dysphagia diets [166]. Specifically, thermo-responsive oleogels undergo reversible structural transitions at specific temperatures to control flavor release and protect thermosensitive bioactives, while pH-responsive oleogels disassemble in gastrointestinal environments to enable targeted nutrient delivery and improve the bioaccessibility of encapsulated ingredients. For instance, a previous study developed novel pH- and thermal-responsive oleogel capsules with a core–shell structure and high monodispersity, prepared via a gravity-assisted co-flowing microfluidic device and simple air-drying. Looking ahead, multi-responsive oleogels that integrate temperature, pH, ionic strength, and enzyme responsiveness are expected to enable dynamic functional regulation throughout food processing, storage, and digestion. Shi et al. [164] developed novel pH- and thermal-responsive core–shell oleogel capsules with high monodispersity prepared via a gravity-assisted co-flowing microfluidic device and simply air-drying. In the future, multi-responsive oleogels integrating temperature, pH, ionic strength and enzyme response are expected to realize dynamic functional regulation throughout food processing, storage and digestion. Nevertheless, their responsive molecular mechanisms remain unclear and the development of food-grade responsive gelators is still immature. Therefore, further research is required to clarify the structure–response–function relationships and verify their practical applicability, which will support the innovation of intelligent functional foods and personalized nutrition systems.

## 5. Conclusions

Edible oleogels represent attractive lipid structuring platforms capable of converting liquid oils into semi-solid materials. This approach helps lower dietary saturated fat consumption and avoids industrially produced trans fats. Their development has been supported by advances in oleogelators and processing technologies, which have expanded their potential applications in food systems. However, most current studies remain at the formulation and laboratory validation stage, and real industrial adoption is still limited.

The functionality of oleogels is governed by a hierarchical structure spanning molecular, microscopic, and macroscopic levels. Molecular interactions drive gelator self-assembly, which further develops into crystalline or aggregated networks that immobilize liquid oil. These structures ultimately determine rheological behavior, texture, and stability. This review identifies three transferable design principles. First, oil composition affects oleogel performance primarily through its compatibility with the oleogelator, its minor components, and its influence on crystallization rather than through a simple saturated-versus-unsaturated distinction. Second, multi-component formulations are advantageous only when complementary assembly mechanisms generate a network response that exceeds concentration-matched single-component controls. Third, processing history is an integral design variable because cooling, shear, and ultrasonication determine whether a given formulation develops a fine interconnected network or a weak, heterogeneous structure.

From an application perspective, oleogel selection should be requirement-driven. Crystalline wax and monoglyceride networks are efficient oil immobilizers but may be brittle or polymorphically unstable; ethylcellulose provides thermal resistance at the cost of high processing temperatures; and biopolymer-based indirect systems support clean-label design but introduce moisture sensitivity and process complexity. These trade-offs explain why results are not directly transferable among bakery, meat analog, frying, and bioactive-delivery applications.

Future progress therefore depends on standardized reporting, quantitative comparison with appropriate controls, and predictive models linking oil composition, molecular interactions, processing history, network architecture, and end-use performance. Sustainable food-grade oleogelators and scalable manufacturing remain important, but industrial translation will require simultaneous optimization of functionality, sensory quality, oxidative stability, safety, cost, and regulatory compliance.

## Data Availability

No new data were created or analyzed in this study.
